# E3 ubiquitin ligases in the acute leukemic signaling pathways

**DOI:** 10.3389/fphys.2022.1004330

**Published:** 2022-11-11

**Authors:** Qianru Zhan, Heyang Zhang, Boquan Wu, Naijin Zhang, Lijun Zhang

**Affiliations:** ^1^ Department of Hematology, The First Hospital of China Medical University, Shenyang, China; ^2^ Department of Cardiology, The First Hospital of China Medical University, Shenyang, China

**Keywords:** E3 ubiquitin ligases, acute leukemia, Notch, JAK2, NF-κB, PI3K/AKT, Wnt/β-catenin

## Abstract

Acute leukemia is a common hematologic tumor with highly genetic heterogeneity, and many factors are involved in the pathogenesis and drug-resistance mechanism. Emerging evidence proves that E3 ubiquitin ligases participate in the acute leukemic signaling pathways *via* regulating substrates. This review summarized the E3 ligases which can affect the leukemic signal. It is worth noting that the abnormal signal is often caused by a deficiency or a mutation of the E3 ligases. In view of this phenomenon, we envisioned perspectives associated with targeted agonists of E3 ligases and proteolysis-targeting chimera technology. Moreover, we emphasized the significance of research into the upstream factors regulating the expression of E3 ubiquitin ligases. It is expected that the understanding of the mechanism of leukemic signaling pathways with which that E3 ligases are involved will be beneficial to accelerating the process of therapeutic strategy improvement for acute leukemia.

## Introduction

Acute leukemia is a hematopoietic stem cell malignancy that is highly heterogeneous. Most abnormally proliferating cells are primitive and naïve cells due to the stagnation of leukocyte differentiation in the early stage. According to the type of cells involved, acute leukemia can generally be divided into two major groups: acute myeloid leukemia (AML), characterized by an uncontrolled clonal proliferation of abnormal myeloid stem/progenitor cells, and acute lymphoblastic leukemia (ALL), characterized by abnormal proliferation and aggregation of immature lymphocytes in the bone marrow and extramedullary tissue. The common clinical manifestation of acute leukemia presented with anemia, hemorrhage, fever, hepatomegaly, splenomegaly, or enlarged lymph nodes. Formal diagnosis has progressed to dimensional of MICM. This means the combination of morphology, immunology, cytogenetics, and molecular biology (Chang et al., 2021; Newell and Cook, 2021). Leukemogenesis is the result of the interaction of various complex mechanisms, among which chromosomal aberrations and gene mutations play an important role. Identifying the molecular abnormalities contributes to formulating risk stratification and improving treatment strategies. At present, some breakthroughs have been achieved, such as all-trans retinoic acid targeted PML-RARα fusion gene and Imatinib targeted BCR-ABL fusion gene; Chimeric Antigen Receptor T-Cell Immunotherapy (CAR-T) targeted CD19; and Gemtuzumab ozogamicin targeted CD33-positive AML (Baron and Wang, 2018; Zhu and Gao, 2019; Levin et al., 2021; Yilmaz et al., 2021). Despite this, the enhancement of overall survival in patients with leukemia remains challenging, and a good knowledge of the acute leukemic signal transduction involved would bring a significant improvement in treatment regimens.

**FIGURE 1:**
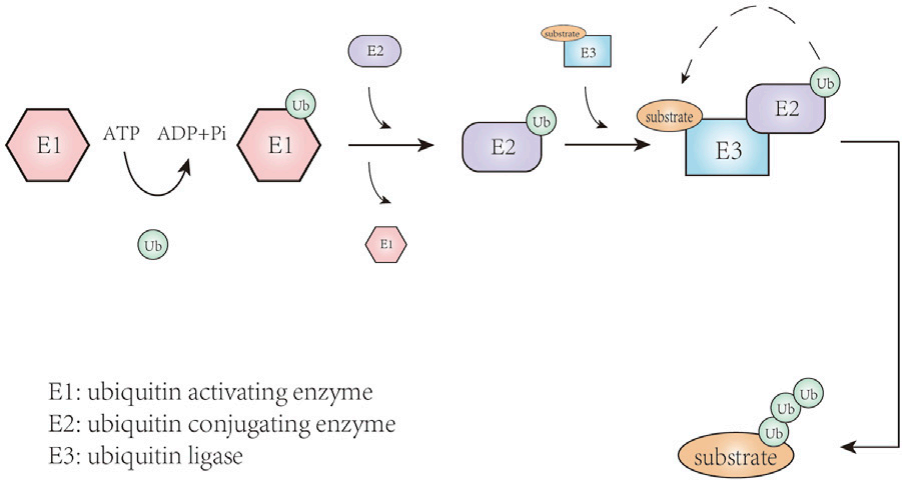
The process of ubiquitination.

Protein translation modifications are one of the most important regulatory mechanisms for intracellular proteins, enabling cells to respond to changes in the internal and external environment through rapid and reversible modifications to the structure, function, and location of a specific protein. More than two hundred protein translation modifications have been discovered, including phosphorylation, methylation, acetylation, ubiquitination, to name a few, which play an important role in cell growth, metabolism, differentiation, apoptosis, and other processes (Dunphy et al., 2021). Ubiquitination refers to the covalent binding of ubiquitin to a target protein (Figure 1), a process that involves the synergistic action of three ubiquitin enzymes: E1 ubiquitin activating enzyme, E2 ubiquitin conjugating enzyme, and E3 ubiquitin ligase (Nandi et al., 2006). Ubiquitin is a 76-residue small protein in which the C-terminal cysteine is activated by E1 ubiquitin activating enzyme and then transferred onto the activate site of an E2 ubiquitin conjugating enzyme through trans-thioesterification (Mansour, 2018; Wijk et al., 2019). Subsequently, E2-ubiquitin intermediate is linked to the target protein by E3 ubiquitin ligases-mediated isopeptide bond formation between the C-terminal glycine of ubiquitin and the substrate lysine residue. In this series of enzymatic cascade reactions, E3 plays a unique role in recognizing target proteins and regulating ubiquitination system activity. E3 ubiquitin ligases fall into three main types: the really interesting new gene/U (UFD2)-box (RING/U-box), the homologous to E6AP carboxyl-terminus (HECT), and the RING-between-RING (RBR) families (Berndsen and Wolberger, 2014; Wang et al., 2017). These E3 ligases can target a variety of substrates, then trigger ubiquitination and proteasome degradation. When this protein degradation process is out of balance due to changes in the E3 ligases, a variety of diseases can be caused or promoted, such as neurodegenerative disorders, cardiovascular disease, and cancer.

**FIGURE 2:**
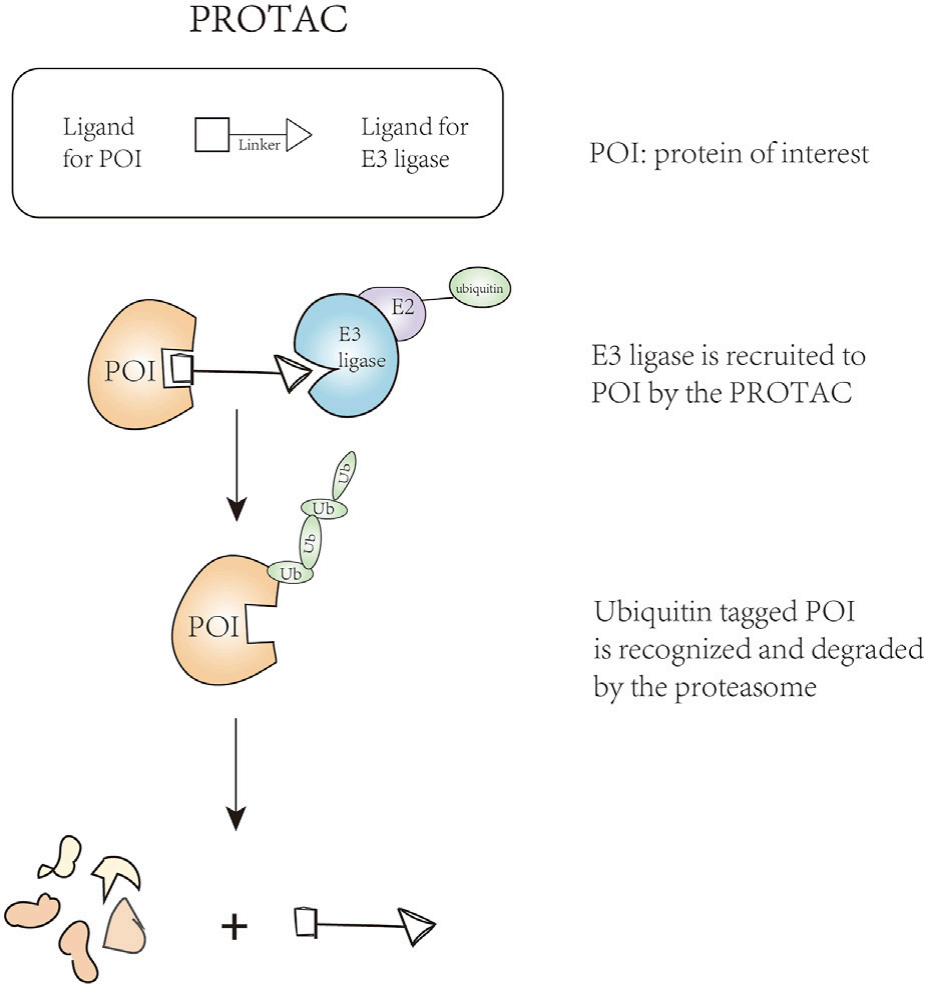
The technique of proteolysis-targeting chimeras.

With the development of proteolysis-targeting chimeras (PROTAC), E3 ligases have become the key target. This technique can remove unwanted or damaged proteins by forming a stable target protein/PROTAC/E3 ternary complex (Figure 2), solving the problem of undruggable cases to some extent (Sakamoto et al., 2001; Moon and Lee, 2018). In addition to PROTAC, there are targeted inhibitors of E3 ligases, targeted agonists of E3 ligases, and molecular glues, thereby arousing our interest in the mechanism of E3 ubiquitin ligases in the acute leukemic signaling pathways. Combined with these techniques, E3 ligases can be excellent regulatory targets affecting anti-leukemia potential. In this review, we summarized E3 ligases that their changes are involved in the abnormal activated signaling pathway, ultimately promoting the occurrence or progression of leukemia.

## The structure and function of E3 ligases involved

### RING-type E3 ubiquitin ligases

RING family is the most common E3 ligases; the RING domain of RING E3s harbors two zinc ions, providing Zn-coordination in a cross-braced configuration for domain folding (Das et al., 2021). RING E3s exerted their E3 activity with a highly diverse quaternary architecture and different modes of assembly: monomer, homodimer, heterodimer, and compositions of multiple subunits (also known as higher order oligomers) (Buetow and Huang, 2016). Additionally, the U-box domain is the same as the RING fold, but without zinc.

The tumor necrosis factor receptor-associated factor (TRAF) family consists of five members: TRAFs 1, 2, 3, 5, and 6, participating in cell proliferation, differentiation, survival, apoptosis, immune and inflammatory responses. Among these members, TRAF2 and TRAF6 have a typical TRAF structure, including an N-terminal RING domain, four or five zinc finger motifs that provide structural support for RING domain activity, and a C-terminal domain that consists of a coiled coil domain and a TRAFC domain (also known as the MATH domain) (Table 1) (Lamothe et al., 2008; Arkee and Bishop, 2020). Besides, the common functional domains of the Casitas B-cell lymphoma (CBL) family (mainly c-Cbl and Cbl-b) include the tyrosine kinase binding domain consisting of four helix bundles, an EF hand, and an Src homology 2 (SH2) domain, which is responsible for recognition of the substrate; a highly conserved helical linker; and one RING finger domain that can work together with the helical linker to form the structural platform for binding to an E2 ubiquitin conjugating enzyme (Table 1) (Meng et al., 1999; Zheng et al., 2000). Cbl-b is a larger protein than c-Cbl and contains an additional 69 amino acids at the C-terminus (Lyle et al., 2019). Specific substrates of CBL are epidermal growth factor receptor (EGFR), platelet-derived growth factor receptor (PDGFR), c-Kit, FLT3, ZAP70 and SYK (Thien and Langdon, 2005; Mohapatra et al., 2013). In addition, the tripartite motif containing (TRIM) proteins contain a large family of RING type E3 ligases. There are three common structural features of TRIMs, containing the N-terminal catalytic RING domain, one or two B-boxes, and a coiled-coil domain (Table 1) (Meroni and Diez-Roux, 2005). Functionally, it has been identified that TRIM56 and TRIM65 can stimulate type I interferon expression combined with nucleic acid-sensing receptors, such as STING, RIG-I, and MDA5 (Tsuchida et al., 2010; Versteeg et al., 2013; Kamanova et al., 2016). Furthermore, TRIAD1 (Two RING fingers and DRIL) is a cysteine-rich domain of around 200 amino acids, consisting of a DRIL motif located between two RING fingers (Reijden et al., 1999). The N-terminal is an acid domain while the C-terminal region contains a helical ring domain (Table 1). TRIAD1 is a proapoptotic protein that promotes p53 activation that plays a critical role in cell differentiation and apoptosis and is ubiquitinated by Mouse double minute 2 (MDM2) (Marteijn et al., 2005; Bae et al., 2012). MDM2 is a p53-specific E3 ubiquitin ligase, acting to limit the p53 growth-suppressive function in unstressed cells (Moll and Petrenko, 2003). MDM2 N-terminal domain is responsible for recognizing and binding to substrates; the C-terminal domain catalysis the transfer of ubiquitin to substrates (Table 1) (Rotin and Kumar, 2009). MDM2 expression is up-regulated in numerous cancers, resulting in a loss of p53-dependent activities, such as apoptosis and cell-cycle arrest (Oliner et al., 2016).

**TABLE 1:** The structure of E3 ubiquitin ligases discussed in this work.

Enzymes	Family	Structure and function
TRAF Lamothe et al., (2008); Arkee and Bishop, (2020)	RING	• N-terminal RING domain
		• four or five zinc finger motifs that provide structural support for RING domain activity
		• C-terminal domain that consists of a coiled coil domain and a TRAFC domain
CBL Meng et al., (1999); Zheng et al., (2000)	RING	• N-terminal tyrosine kinase binding domain consists of four helix bundles, an EF hand, and an Src homology 2 (SH2) domain, which is responsible for recognition of the substrate
		• a highly conserved helical linker
		• C-terminal RING finger domain that can work together with the helical linker to form the structural platform for binding to an E2 ubiquitin conjugating enzyme
TRIM Meng et al., (1999); Zheng et al., (2000)	RING	• N-terminal catalytic RING domain
		• one or two B-boxes
		• a coiled-coil domain
TRIAD1 Reijden et al., (1999); Marteijn et al., (2005)	RING	• N-terminal is an acid domain
		• a DRIL motif located between two RING fingers
		• two C-terminals are the helical ring domain
MDM2 Rotin and Kumar, (2009)	RING	• N-terminal domain is responsible for recognizing and binding to substrates
		• C-terminal domain catalysis the transfer of ubiquitin to substrates
SKP2 Bai et al., (1994); Nguyen and Busino, (2020)	F-box	• substrate receptor of Cullin-RING
		• a class of proteins contain a sequence homologous to cyclin F and can catalyze the substrate by binding to SKP1 and CUL1
FBW7 Hao et al., (2007); Tang et al., (2007)	F-box	• substrate receptor of Cullin-RING
		• the D domain is mainly responsible for FBW7 dimerization, regulating substrate binding modes and ubiquitylation
		• F-box domain is an essential part for FBW7 combining to SKP1
		• seven tandem WD40-repeat domains play a role in phosphorylated substrates recognition
ULF Chen et al., (2010a)	HECT	• N-terminal ARM domain is responsible for recognizing and binding to substrates
		• a centrally located WWE motif
		• C-terminal HECT domain catalyzes the transfer of ubiquitin to substrates
WWP1 Hu et al., (2021)	HECT	• N-terminal is a C2 domain
		• four WW domains in its central part
		• C-terminal is a catalytic HECT domain

### F-box E3 ubiquitin ligases

F-box protein functions as the substrate receptor in Cullin-RING E3 ubiquitin ligases (CRLs), which consists of three categories: FBXWs, FBXLs, and FBXOs. Among the RING-type E3 ligases, the Cullin is the largest family. CRLs belong to RING-type E3s with a characterized Cullin-RING heterodimeric complex, containing seven Cullin (CUL) proteins, CUL1, CUL2, CUL3, CUL4A, CUL4B, CUL5, and CUL7. The core structure of CRL consists of four parts: the CUL scaffold, a substrate receptor, adaptor proteins that connect the substrate receptor to CUL, and a RING finger protein binding to E2 ubiquitin conjugating enzyme. The C-terminal domain of CUL protein forms a core ligase complex in combination with a RING finger protein, either RBX1 or RBX2, while the N-terminal domain interchangeably assembles with CUL-specific substrate receptors through adaptors. For instance, CRL1, also named SKP1–CUL1–F-box (SCF), uses SKP1 as its adaptor, and F-box as substrate receptor (Skowyra et al., 1997; Nakagawa et al., 2020).

S-phase kinase-associated protein 2 (SKP2), which belongs to F-box protein is a class of proteins containing a sequence homologous to cyclin F and can catalyze the substrate by binding to SKP1 and CUL1 (Bai et al., 1994; Jin et al., 2004; Nguyen and Busino, 2020). The CRL1^SKP2^ consists of four components: CUL1, SKP1, the F-box domain of SKP2 (F-box^SKP2^), and RBX1 (Table 1). Functionally, SKP2 targets various cyclin-dependent kinases inhibitors (CKI) for degradation, such as p21^Cip1^, p27^Kip1^, and p57^Kip2^, controlling cell cycle regulatory proteins (Frescas and Pagano, 2008). Therefore, the dysfunction of SKP2 causes cell cycle entry or arrest. F-box and WD-40 repeat domain-containing protein 7 (FBW7), a component of an SCF ubiquitin ligase complex, belongs to the F-box protein family. The structure of FBW7 is organized in three domains: the D domain, F-box domain, and seven tandem WD40-repeat domains. The D domain is mainly responsible for FBW7 dimerization, regulating substrate binding and ubiquitylation; the F-box domain is essential for FBW7 interaction with SKP1 whereas the WD40-repeat domain plays a role in the recognition of phosphorylated substrates (Table 1) (Hao et al., 2007; Tang et al., 2007). The classical substrates of FBW7 include c-Myc (Yada et al., 2004) and cyclin-E (Koepp et al., 2001), although other target proteins have been identified such as neurogenic locus Notch homolog protein 1 (Notch 1) (Fryer et al., 2004), NF-κB (Fukushima et al., 2012), MCL-1 (Wertz et al., 2011), c-Jun (Wei et al., 2005), granulocyte colony stimulating factor receptor (Lochab et al., 2013), heat shock transcription factor 1 (Kourtis et al., 2015), CCAAT/enhancer-binding protein-alpha (C/EBPα) (Bengoechea-Alonso and Ericsson, 2010), and glucocorticoid receptor α (Malyukova et al., 2013). Additionally, the expression of FBW7 is regulated by a series of genes, including p53 (Kimura et al., 2003), miR-223 (Mansour et al., 2013), miR-25 (Xiang et al., 2015), miR-182 (Li et al., 2014), miR-503 (Li et al., 2014), miR-92a (Zhou et al., 2015), RBP-J-interaction, tubulin-associated (RITA) protein (Wang et al., 2014), NF-κB1 (Huang et al., 2014).

### Homologous to E6AP carboxyl-terminus-type E3 ubiquitin ligase

Different from the scaffolding role (combining E2 closely with substrates) of RING-type E3 ligases, HECT-type E3 ligases play a catalytic role. For the HECT E3 ligase, it consists of an N-terminal substrate-binding domain and a C-terminal HECT domain. The C-terminal HECT domain was first discovered in human papillomavirus E6-associated protein (E6AP) 5, containing almost 350 amino acids (Rotin and Kumar, 2009; Singh and Sivaraman, 2020); there are two lobes in the conserved HECT domain that are connected by a flexible hinge loop, with the N-terminal lobe (N-lobe) binding to E2∼ubiquitin and the C-terminal lobe (C-lobe) having the catalytic cysteine residue (Huang et al., 1999). Based on the different N-terminal domains, HECT E3s can be divided into NEDD4 family, HERC family, and HECTs with other protein-protein interaction domains (Singh et al., 2021). Ubiquitin ligase for ARF (ULF) was identified in nucleophosmin (NPM) protein complexes. It includes an N-terminal ARM domain and a C-terminal HECT domain, with a centrally located WWE motif (Table 1) (Chen et al., 2010a). The main function of ULF is to mediate the polyubiquitination and proteasomal degradation of alternative reading frame (ARF) protein (Chen et al., 2010a). As a member of the NEDD4 family, WW domain-containing E3 ubiquitin protein ligase 1 (WWP1) composes a C2 domain in N-terminal and a catalytic HECT domain in C-terminal, with four WW domains in its central part (Hu et al., 2021). Functionally, WWP1 interacts with a variety of substrates, such as TβR1 (W1Komuro et al., 2004), Smad2 (Chen et al., 2007), ErbB4/HER4 (Feng et al., 2009), RNF11 (Chen et al., 2008), SPG20 (Edwards et al., 2009), RUNX2 (Shen et al., 2006), p63 (Li et al., 2008), and p27 (Cao et al., 2011). These interactions regulate numerous physiology processes, including TGF signaling, osteoblast differentiation, differentiation and apoptosis of cancer cells, senescence.

## Effects of E3 ubiquitin ligases on Notch signaling

**FIGURE 3:**
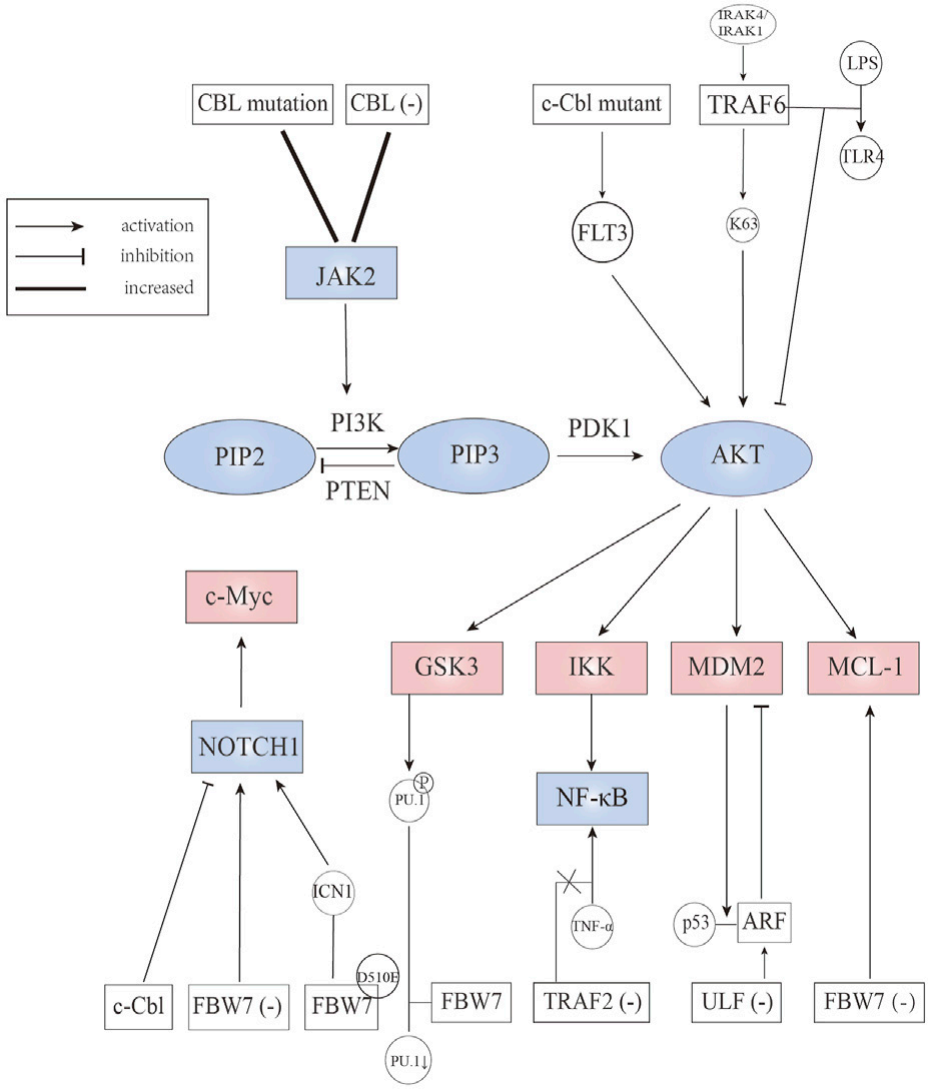
E3 ubiquitin ligases involved in JAK2, Notch, PI3K/AKT, and downstream signaling pathways.

FBW7 is deemed to be a p53-dependent tumor suppressor (Kimura et al., 2003; Mao et al., 2004). In the incidence of T-ALL, FBW7 deletion without other tumor-promoting factors is one of the causes (Matsuoka et al., 2008). Evidence showed that 59% FBW7 deletion mice developed T-ALL, along with Notch1 and c-Myc proteins accumulated in leukemic cells while p53 protein level was decreased. Notch1 is a transmembrane protein that serves as a ligand-activated transcription factor. The Notch1/c-Myc signaling pathway plays a pivotal role in T-ALL (Weng et al., 2004; Weng et al., 2006). FBW7 mutations, which are mainly located in codons that encode for arginine residues R479Q, R505C, and R465H, have been identified in T-ALL, accounting for 31% of all T-ALL cases (O’Neil et al., 2007; Akhoondi et al., 2007). It is worth noting that spontaneously developed leukemia was not seen in mice that carry Cre-inducible FBW7 heterozygote mutants (King et al., 2013). Notch1 mutation, as an important oncogenic factor in ALL, is also associated with FBW7 mutation. Notch1 mutations mainly occur in the PEST domain and the heterodimeric domain. The reason for the name PEST is that this mutation truncates the C-terminal domain of protein, among which the proline, glutamine, serine, and threonine have a high frequency of truncation and are known as PEST domains (Chiang et al., 2016). Mechanistically, disruption or deletion of the PEST domain can be found in 15% T-ALL and has a relationship with FBW7 mutation, causing increased intracellular Notch1 (ICN1) protein stabilization; heterodimeric domain point mutation can be found in 25% T-ALL and has a relationship with PEST or FBW7 mutations, contributing to ligand-independent activation of the receptor (Weng et al., 2004). ICN1 splits from the cell membrane and migrates into the nucleus. The extended half-life of ICN1 causes sustained activation of Notch1 signaling pathway, ultimately leading to blocking of apoptosis in normal cells and the abnormal proliferation of non-functional T cells. Adult T cell leukemia/lymphoma (ATL), an aggressive lymphoproliferative disease with poor prognosis, is caused by the human T-cell leukemia virus type 1 (HTLV-1). In HTLV-1-transformed ATL cells, FBW7 acts as a tumor suppressor as well. According to the study of O’Neil et al. (2007), the FBW7 mutation, which is located in the beta-propeller domain adjacent to the pocket for substrate binding, activates the Notch1 signaling pathway. To be more specific, FBW7 mutants T416A and W406R retain the ability to interact and degrade ICN1, while the majority of FBW7 mutants lose that ability. In contrast to T416A and W406R mutants, the FBW7 mutants D510E can normally interact with ICN1, but lose its degradation ability, leading to the continued activation of the Notch1 signaling pathway (Figure 3). Interestingly, FBW7 D510E can target c-Myc, cyclin E, and MCL-1. The reason why FBW7 D510E selectively degrades substrates remains unknown. In addition, hyperphosphorylation of cyclin E, which is related to the FBW7 mutation, is highly correlated with polyploidy and aneuploidy (Table 2). The latter is one of the characteristics of acute ATL (Rajagopalan et al., 2004).

**TABLE 2:** The regulation between E3 ligases and leukemic signaling pathways.

Enzymes	Leukemic signaling pathways	Function	Disease or cell type
TRAF6	PI3K-AKT Chan et al., (2012); Schnetzke et al., (2013a); Li et al., (2015)	• inhibiting AKT activation after stimulation of TLR-4 with lipopolysaccharide	FLT3-ITD AML
		• IRAK4/IRAK1 induces TRAF6 activation and then activates AKT through K63 polyubiquitination, stabilizing MCL-1	primary T cells
	NF-κB Lomaga et al., (1999); Verstrepen et al., (2008); Schnetzke et al., (2013a)	• activation of NF-κB following stimulation of lipopolysaccharide-mediated TLR-4 activation	—
		• TRAF6 plays a pivotal role in TLR signal and is involved in the NF-κB pathway	
TRAF2	NF-κB Chung et al., (2007); Schnetzke et al., (2013b)	• playing an important role in NF-κB activation by TNF-α stimulation	—
		• knock-down of TRAF2 makes cells more susceptible to TNF-α	FLT3-ITD AML
SKP2	Notch/SKP2/p27^Kip1^ Zhang et al., (2000); Barata et al., (2001); Dohda et al., (2007); Rodriguez et al., (2020)	• SKP2 genetic ablation can delay T-ALL progression *in vivo*	T-ALL
		• pharmacological blockade of SKP2 can inhibit the proliferation of human T-ALL cells	
		• This arises because SKP2 can degrade the cyclin-dependent kinase inhibitor p27^Kip1^, inducing T cell progression into the cell cycle and coordinating cell proliferation and cell differentiation	
FBW7	Notch1 Matsuoka et al., (2008); Weng et al., (2004); O’Neil et al., (2007); Akhoondi et al., (2007); Rajagopalan et al., (2004)	• FBW7 deletion mice develop T-ALL, along with Notch1 and c-Myc proteins accumulated while p-53 protein level was decreased	T-ALL
		• FBW7 can induce the ubiquitination and degradation of ICN1	
		• Notch1 mutation is associated with FBW7 mutation, which (R479Q, R505C, R465H) was detected in T-ALL	ATL
		• FBW7 mutation (except T416A, W406R) activates the Notch1 signaling pathways	
		• FBW7 mutation is associated with cyclin E hyperphosphorylation and aneuploidy; aneuploidy is one of the characteristics of acute ATL	
	GSK3β Schmidt et al., (2006); Wertz et al., (2011); Mishra et al., (2021)	• promoting degradation of glucocorticoid receptor α through a GSK3β phosphorylated degron	—
		• promoting degradation of MCL-1 in a GSK3-dependent manner	AML
		• FBW7 degrades PU.1 in a GSK3β phosphorylated manner	
CBL	Notch1 Zhu et al., (2020)	• c-Cbl was supposed to facilitate the ubiquitination and degradation of Notch1	T-ALL
		• flavone induces ICN1 degradation through up-regulation of the level of c-Cbl	
	Notch3 Checquolo et al., (2010)	• Notch3 can be degraded in a c-Cbl-dependent manner	
	PI3K-AKT Rathinam et al., (2010); Taylor et al., (2015)	• c-Cbl^A/-^ mice developed aggressive AML under the existence of MPD	AML
		• c-Cbl mutant protein elevates FLT3 signaling resulting in activation of AKT pathway	
		• PDK1 blocks CBL-b and then prevents PI3K degradation, resulting in AKT activation	
	p53 Park et al., (2017)	• FLT3-ITD AML cells could inactivate p53, resisting the effects of FLT3 inhibitors	
	JAK2 Lv et al., (2017)	• knock-down of CBL can prolong JAK2 activation	
		• CBL mutation can extend the half-life of JAK2	
		• CBL mutant AML cells are more sensitive to JAK2 inhibitor ruxolitinib	
ULF & MDM2	MDM2-p53 Weber et al., (1999); Llanos et al., (2001); Chen et al., (2010a)	• knock-down of ULF increases the level of ARF protein	—
		• ARF binds to and suppresses the activity of MDM2, contributing to p53 activation	
TRIM56	Wnt/β-catenin Yan et al., (2021)	• NEAT1 can enhance the degradation of DVL2 by TRIM56, then inactivating the Wnt signaling pathway	AML
TRIAD1	RTK Wang et al., (2018); Wang et al., (2020)	• TRIAD1-substrate RTKs inhibitor terminated emergency granulopoiesis, delayed leukemogenesis during emergency granulopoiesis	MLL-AML
WWP1	Autophagy Sanarico et al., (2018)	• WWP1 depletion can transform LC3-I to LC3-II accompanied by the accumulation of ATG7	—
		• the level of SQSTM1/p62 was decreased	

In T-ALL, ICN1 is the potential therapeutic target, an aspect that is described in detail in the section covering FBW7. However, although γ-secretase inhibitors (GSI) can prevent the release of ICN1, they also lead to off-target effects. As for c-Cbl (a member of the CBL family), such mutation is frequent in myeloid leukemia but not in T-ALL, making it a possible drug target. Recent studies have shown that flavone (2-phenyl-4H-1-benzopyran-4-one), the core structure of flavonoids, can induce ICN1 degradation through up-regulation of the level of c-Cbl, inhibiting cell proliferation (Zhu et al., 2020). In addition to Notch1, Notch3 was identified as another member of the Notch family that plays a critical role in the development of T-ALL. In the absence of pTα, Notch3 can be degraded in a c-Cbl-dependent manner (Checquolo et al., 2010). Furthermore, SKP2 was reported to play a crucial role in T-ALL. Recent research advances demonstrated that SKP2 genetic ablation can delay T-ALL progression *in vivo*, while the pharmacological blockade of SKP2 can inhibit the proliferation of human T-ALL cells (Rodriguez et al., 2020). This arises because SKP2 can degrade the cyclin-dependent kinase inhibitor p27^Kip1^, inducing T cell progression into the cell cycle and coordinating cell proliferation and cell differentiation. Moreover, it has been reported that Notch activation promotes the role of SKP2 in T-ALL, forming a Notch/SKP2/p27^Kip1^ forward feedback loop (Table 2) (Zhang et al., 2000; Barata et al., 2001; Dohda et al., 2007; Rodriguez et al., 2020).

## Effects of E3 ubiquitin ligases on janus kinase 2 signaling

Cytokine dependent Janus kinase 2 (JAK2) signaling is crucial to hematopoietic stem/progenitor cells. Uncontrolled activation of JAK2 signal triggers the occurrence of hematological malignancy. A study assessed whether CBL influence JAK2 protein levels showed that CBL knock-down can prolong JAK2 activation (Lv et al., 2017). Similarly, an elevated level and extended half-life of JAK2 was observed in CBL mutant AML cells. However, independent studies have shown that compared to CBL wild-type AML cells, CBL mutant AML cells are more sensitive to quizartinib (a FLT3 inhibitor) than ruxolitinib (a JAK2 inhibitor) (Lv et al., 2017). As c-Cbl mutant protein can stimulate FLT3 signaling, we envisioned the existence of a c-Cbl, JAK2, and FLT3 signaling axis, regulating the development of AML (Figure 3) (Table 2) (Rathinam et al., 2010; Taylor et al., 2015).

## Effects of E3 ubiquitin ligases on phosphatidylinositol 3-kinase/AKT signaling

Phosphatidylinositol 3-kinase (PI3K)/Protein Kinase B (PKB, also named AKT) is known to be associated with cell metabolism, proliferation, differentiation, and apoptosis. The interaction between PI3K and AKT is related to two metabolites and two coding genes: the small molecules PI (4,5) P2 and PI (3,4,5) P3 and the proteins phosphatase and tensin homologue (PTEN) and 3-phosphoinositide dependent protein kinase-1 (PDK1). In normal cells, PI (3,4,5) P3 is rapidly metabolized and dephosphorylated by lipid phosphatases (such as PTEN) to terminate the PI3K signal (Vara et al., 2004). Large deposits of PI(3,4,5)P3 or the loss-of-function of PTEN are often found in cancer cells, suggesting that abnormal regulatory genes play an important role in cancer. In addition, an increasing number of studies show that PI3K/AKT regulates the occurrence and development of leukemia and drug resistance (Nepstad et al., 2020; Yao et al., 2021).

Currently, the link between TRAF6 and AML is widely studied. TRAF6 is considered to play an essential role in signal transduction of the Toll-like-receptor (TLR) superfamily (classified as immune receptors). TRAF6 inhibits the activation of AKT after stimulation of TLR-4 with lipopolysaccharide in FLT3-ITD AML cells (Schnetzke et al., 2013a). Down-regulation of TRAF6 results in enhanced constitutive AKT activation through phosphorylating residues Thr308 and Ser473 in MV4-11 cells (FLT3-ITD AML cells). Interestingly, Chan et al. (2012) suggested that the ubiquitination and activation of AKT were affected by diverse growth factors utilizing distinct E3 ligases. For example, the SKP2-SCF complex was required for EGFR induced AKT activation; SKP2 underwent tyrosine and serine/threonine phosphorylation after EGF stimulation. TRAF6 not only acts in AML, but also in ALL. In primary T cell, TRAF6 was activated through MyD88/IL-1 receptor-associated kinase (IRAK) 4/IRAK 1 signaling, then catalyzing K63 polyubiquitination (which is known to activate AKT directly), and ultimately stabilizing antiapoptotic protein MCL-1 (Figure 3) (Li et al., 2015). The study also showed that inhibition of IRAK reduced the stability of MCL-1 and enhanced the sensitivity to chemotherapy (Table 2) (Li et al., 2015).

Deletion and loss-of-function mutation of c-Cbl can be found mainly in myeloproliferative neoplasms (MPNs) but also occured in other hematopoietic malignancies such as AML (Dunbar et al., 2008). AML patients with CBL mutation always need an intense chemotherapy regimen or accept hemopoietic stem cell transplantation, showing highly malignant characteristics (Makishima et al., 2009). Studies in animal models have shown that single copy mutant c-Cbl^A^/^−^ mice developed aggressive myeloid leukemia with significantly increased white blood cell counts. The stimulation of FLT3 signaling caused by c-Cbl mutant protein results in the constitutive activation of the AKT pathway (Figure 3) (Table 2), suggesting that FLT3 kinase is a potential therapeutic target for the treatment of c-Cbl mediated leukemia (Rathinam et al., 2010; Taylor et al., 2015). In this regard, it is worth mentioning a recent study showing that Cbl-b was implicated in FLT3 kinase inhibitor-resistant AML. According to Park et al. (2017), the mutant FLT3-ITD AML cells could inactivate p53, resisting the effects of FLT3 inhibitors.

## Effects of E3 ubiquitin ligases on NF-κB signaling

NF-κB, a generic name of a family of transcription factors, regulates a series of genes involved in cell survival, proliferation, differentiation, and immune and inflammatory responses. Mounting evidence shows that constitutive activation of NF-κB is frequently associated with hematological malignancies. NF-κB selective inhibitory drugs have been developed for targeting tumor cells (Breccia and Alimena, 2010). At present, NF-κB inhibitors such as bortezomib and carfilzomib are some of the potent therapeutically useful drugs for multiple myeloma. Activation of NF-κB signaling can be caused by stimulation of lipopolysaccharide-mediated TLR-4 activation and tumor necrosis factor-α (TNF-α) (Lomaga et al., 1999; Verstrepen et al., 2008; Schnetzke et al., 2013a). Previous studies have shown that TRAF6 is involved in the NF-κB signaling pathway (Min et al., 2018). However, more studies are warranted to ascertain whether TRAF6 activates NF-κB. Besides TRAF6, TRAF2 is of great importance in NF-κB activation by TNF-α stimulation (Chung et al., 2007). The anti-apoptosis effect of NF-κB is part of the reason for TNF-α mediated avoidance of programmed cell death (Ren et al., 2007). In the heterozygous FLT3-ITD positive MOLM-13 cell line, cells presented a higher susceptibility to TNF-α after TRAF2 knock-down compared with control cells, and induction of apoptosis and impaired proliferation after TNF-α exposure were observed (Table 2) (Schnetzke et al., 2013b). This result proves the potential antiapoptotic role of TRAF2.

## Effects of E3 ubiquitin ligases on phosphatidylinositol 3-kinase/AKT downstream signaling

FBW7 promotes ubiquitylation and proteasomal degradation of glucocorticoid receptor α through a conserved, GSK3β phosphorylated degron (Malyukova et al., 2013). Glucocorticoid is an essential part of ALL chemotherapy regimens and glucocorticoid sensitivity can be restored through overexpression of glucocorticoid receptor (Schmidt et al., 2006). Malyukova et al. (2013) confirmed that FBW7 inactivation leads to up-regulation of glucocorticoid receptor and enhanced glucocorticoid sensitivity in glucocorticoid-resistant cells. Additionally, FBW7 targets MCL-1 for ubiquitination and degradation in a GSK3-dependent manner (Wertz et al., 2011). FBW7 deletion can up-regulate the expression of MCL-1 in T-ALL cell line, which is an anti-apoptotic protein of the BCL-2 family, causing increased sensitivity to sorafenib (a tyrosine kinase inhibitor) but decreased sensitivity to ABT-737 (a pan-inhibitor of the Bcl-2 family of anti-apoptotic proteins) (Yang-Yen, 2006; Inuzuka et al., 2011). Furthermore, FBW7 mutation is partly the reason for resistance to GSI, which is effective against T-ALL cell lines harboring wild-type PTEN deletion on chromosome ten (Weng et al., 2004; Hales et al., 2014). GSK3β, a multifunctional serine/threonine kinase that is overexpressed and hyperactivated in AML, is considered a tumor promoter in mixed lineage leukemia (MLL) (Wang et al., 2008). Several studies showed that GSK3β inhibition contributes to differentiation and apoptosis of leukemic cells (Zhou et al., 2011; Gupta et al., 2012; Gupta et al., 2016). Mechanically, in AML cell lines THP1 and U937, both GSK3β inhibition and proteasome inhibition can enhance the expression of Purine Rich Box-1 (PU.1), which is a central regulator of the differentiation of all hematopoietic cell lineages (Dakic et al., 2005; Mishra et al., 2021). Suppression of PU.1 leads to leukemic transformation of myeloid cells, indicating that PU.1 plays a tumor suppressor role in myeloid cells (Metcalf et al., 2006). Further experiments indicated that PU.1 is phosphorylated by GSK3β and then recognized by FBW7, resulting in ubiquitination and degradation (Figure 3) (Table 2) (Mishra et al., 2021). Taken together, these studies suggest that targeting the GSK3-FBW7 signaling axis provides a possibility of inhibiting AML growth and induce myeloid differentiation.

As a tumor suppressor, ARF can work through p53 pathway activation but also in a p53-independent form. For ARF-p53 axis, the activation of p53 involves multiple mechanisms. On the one hand, a nucleolar form of ARF can bind to MDM2 to sequester it in the nucleolus, and nucleoplasmic forms of ARF can suppress the ubiquitin ligase activity of MDM2, contributing to p53 activation and stabilization (Weber et al., 1999; Llanos et al., 2001). MDM2, a RING-type E3 ubiquitin ligase, is the main regulator of p53 (Vousden and Prives, 2005). On the other hand, ARF can inhibit the enzyme activity of ARF-BP1 (a HECT type E3 ligase), causing p53 stabilization (Chen et al., 2005; Chen et al., 2006). The ubiquitination and degradation of ARF are mediated by ULF, which can directly interact with the C-terminus of NPM to disrupt the protection role for ARF (Chen et al., 2010b). NPM1, one of the most common genetic mutations in AML, can elevate autophagy activity, which contributes to cell survival in NPM1 mutated AML. NPM mutation is found in 35% of AML cells, leading to cytoplasmic dislocation of nucleophosmin (NPM-c) that cannot retain ARF in the nucleoli (Quentmeier et al., 2005). In NPM-c AML cells, ARF is unstable and can be degraded rapidly, whereas ULF knock-down can stabilize ARF and activate the ARF-p53 axis (Figure 3) (Table 2), making ULF an effective target in AML cells (Chen et al., 2010b).

## Effects of E3 ubiquitin ligases on Wnt/β-catenin signaling

**FIGURE 4:**
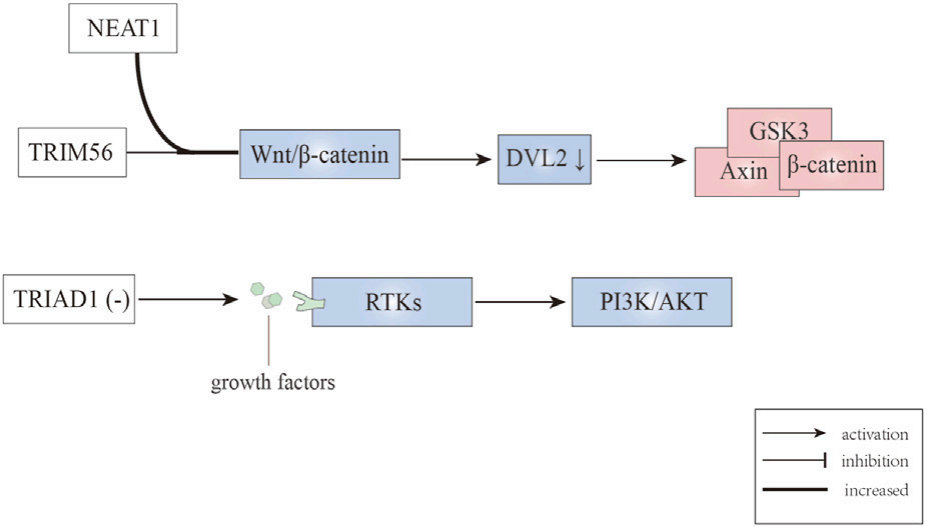
E3 ubiquitin ligases involved in Wnt/β-catenin and RTK signaling pathways.

Wnt/β-catenin signaling is critical for leukemic stem cell maintenance, with dishevelled2 (DVL2) functioning as the core component. DVL2 can disassemble the APC/Axin/CK1α/GSK3β degradation complex under the binding between Wnt ligands and Fzd receptors, leading to β-catenin stabilization; while absence of Wnt ligands made β-catenin degrade in the manner of ubiquitin-proteasome (Nusse and Clevers, 2017). The degradation of β-catenin correlates with the leukemic stem cell, contributing to the promotion of AML (Soares-Lima et al., 2020). Yan et al. (2021) studied the mechanism of nuclear paraspeckle assembly transcript 1 (NEAT1) (which belongs to long non-coding RNAs) in leukemogenesis and leukemic stem cell function, finding that loss of NEAT1_1 promotes murine leukemogenesis. Besides, they confirmed that NEAT1 can enhance the degradation of DVL2 by TRIM56, then inactivating the Wnt signaling pathway. Collectively, these finding indicate that E3 ubiquitin ligase TRIM56 combined with DVL2 participate in the inhibition of the Wnt signaling (Figure 4) (Table 2), in a process that is mediated by NEAT1_1.

## Effects of E3 ubiquitin ligases on RTK signaling

Receptor tyrosine kinases (RTKs), which consists of about twenty subfamilies, are implicated in regulating pathways for cell growth, differentiation, adhesion, and cell death. The RTKs commonly referred to as type III, which include c-Kit, CSF1R, FLT3, and PDGFR, have a major impact on leukemogenesis and transformation into AML (Carter et al., 2020). The genetic characteristic of MLL is the chromosome 11q23 abnormality, and this type of leukemia is associated with adverse prognosis. Emergency granulopoiesis is known to accelerate leukemogenesis in MLL1-rearranged AML and this occurs in an RTK-dependent manner (Wang et al., 2020). Moreover, it has been reported that in MLL1-ELL AML mice, TRIAD1 expression is decreased, contributing to sustained RTK signaling and failing to terminate emergency granulopoiesis (Figure 4) (Wang et al., 2018; Wang et al., 2020). Using TRIAD1-substrate RTKs inhibitors can terminate emergency granulopoiesis, delay leukemogenesis during emergency granulopoiesis, and normalize innate immune responses when combined with chemotherapy (Table 2) (Wang et al., 2020). In addition, as a RTK inhibitor, nintedanib can reverse activation (phosphorylation) of AKT in MLL1-ELL^+^ LIN^−^ cells that are associated with inhibited GSK3β phosphorylation and β-catenin destabilization (Wang et al., 2020).

## Effects of E3 ubiquitin ligase on autophagy signaling

**FIGURE 5:**
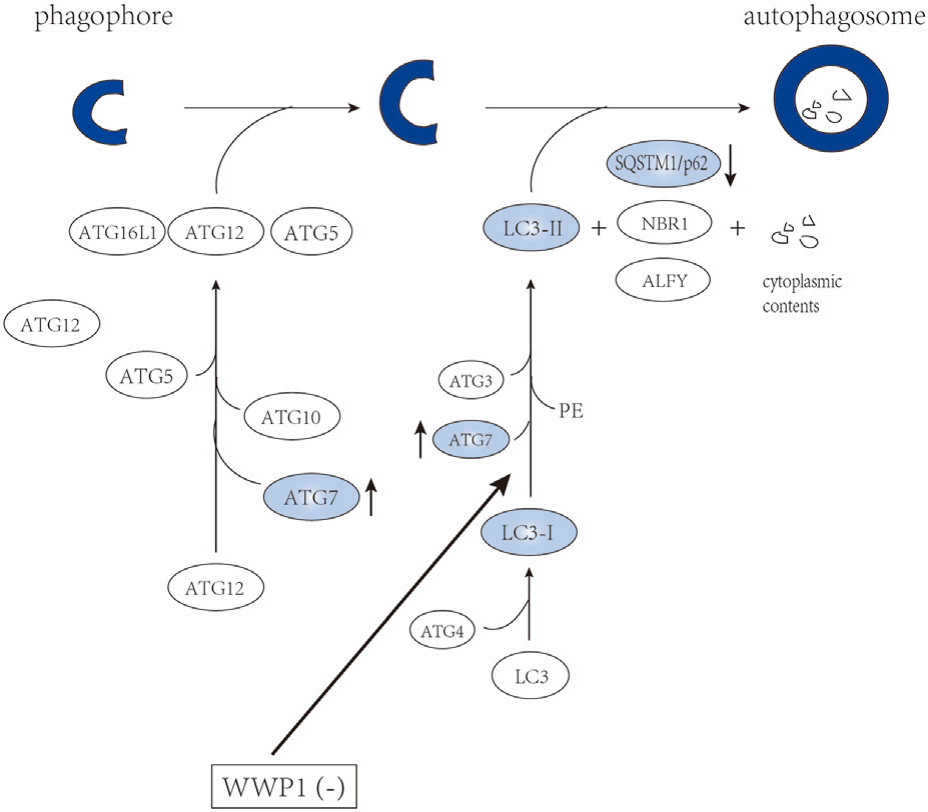
WWP1 involved in maturation of autophagosome.

Autophagy is an intracellular evolutionarily conserved catabolic degradation process, mediated by lysosome sustains (Yu et al., 2018). The process of autophagy is divided into four steps: initiation, nucleation, maturation, and degradation (Feng et al., 2014). The first two steps promote the formation of the autophagic vesicle membrane. During the maturation step, protein conjugation events are necessary for autophagosome formation, involving a series of proteins from the autophagy related gene (ATG) family (Figure 5) (Onorati et al., 2018). LC3-I is formed through the cleavage of LC3 (ATG8) by ATG4. It can conjugate to phosphatidylethanolamine of the autophagosome by ATG3 and ATG7. The level of LC3-II (lipid form of LC3) is proportional to the degree of autophagy (Onorati et al., 2018). According to Sanarico et al. (2018), WWP1 depletion transformed the cytosolic LC3-I to the lipid-bound LC3-II form accompanied by the accumulation of autophagy-associated protein ATG7. Meanwhile, the level of SQSTM1/p62 was decreased, which is a cargo receptor that recruits cargo destined for autophagic degradation to LC3-II on forming autophagasomes (Figure 5) (Sanarico et al., 2018). Overall, WWP1 depletion promotes the autophagic degradation of oncogenic proteins, such as PML-RARα and FLT3-ITD, inducing differentiation of AML cells.

## Conclusion

In conclusion, a line of evidence has highlighted the role of E3 ubiquitin ligases in the abnormal activation of leukemic signaling pathways. An interesting observation is that the absence of the E3 ligases is often the key to causing the abnormal signal. For this situation, targeted agonists of E3 ligases such as CC-90009 represent an advance in AML treatment. CC-90009 can co-opt the CUL4-DDB1-CRBN-RBX1 (CRL4^CRBN^) E3 ubiquitin ligase complex to target G1 to S phase transition 1 (GSPT1) selectively for ubiquitination and proteasomal degradation, inducing AML apoptosis (Hansen et al., 2021). This provides a promising approach to other agonists of E3 ligases. In this regard, it is crucial to clarify what types of factors regulate the expression of E3 ubiquitin ligases because the understanding of upstream mechanisms is beneficial to developing new therapeutic regimens. Additionally, PROTAC can remove unwanted or damaged proteins without the need for a specific target. Whether it is possible to link a normal E3 ligases to a mutant E3 ligase so that the aberrant one can be ubiquitination and proteasomal degradation?
